# Supplementary material for: Skillful multiyear predictions of ocean acidification in the California Current System
Source: Nat Commun. 2020 May 1;11:2166. doi: 10.1038/s41467-020-15722-x (PMC7195403; doi:10.1038/s41467-020-15722-x)
Supplement: Supplementary file 1 — Supplementary Information [file 41467_2020_15722_MOESM1_ESM.pdf]

Supplementary Information for Skillful multiyear predictions of ocean acidification in the California Current System by Brady et al.

Supplementary Table 1. *Correlations between surface variables and climate modes.*

**Variable**	**Pacific Decadal Oscillation (PDO)**	**El Niño–Southern Oscillation (ENSO)**
surface pH	-0.03	-0.07
dissolved inorganic carbon^{+}	0.66*	0.52*
temperature^{+}	-0.73*	-0.64*
alkalinity	-0.22	-0.04
salinity^{+}	-0.20	-0.14
residual	0.43	0.24

Linear correlations are computed between annual averages of variables with a second-order polynomial fit removed. All variables except for surface pH represent the linear response of surface pH to that variable (see Linear Decomposition section in Methods). Asterisks represent statistical significance per *Bretherton et al. 1999*[2] with $\boldsymbol{\alpha = 0.05}$.
$^{+}$An increase in these variables causes increased acidity, or a decrease in surface pH. Thus, a negative correlation coefficient represents a reduction in surface pH, but increase in the environmental variable.

Supplementary Table 2. *Components used in all model experiments.*

Component	Model	Details	Key Reference
Atmosphere	Community Atmosphere Model, version 5 (CAM5)	1° horizontal resolution and 30 vertical levels	Hurrell et al. 2013 [3]
Ocean	Parallel Ocean Program (POP), version 2	1° horizontal resolution and 60 vertical levels	Danabasoglu et al. 2012 [4]
Sea Ice	Community Ice Code (CICE), version 4	Same horizontal grid as the ocean	Hunke and Lipscomb 2008 [5]
Land	Community Land Model (CLM4)	Includes modules for biogeophysics, the hydrological cycle, biogeochemistry, and dynamic vegetation	Lawrence et al. 2011 [6]
Ocean Biogeochemistry	Biogeochemical Elemental Cycling (BEC)	Explicit simulation of carbonate chemistry; three phytoplankton functional types and one zooplankton class; cycling of multiple biogeochemical tracers	Moore et al. 2013 [7]

The components listed here represent those used for the Community Earth System Model Decadal Prediction Large Ensemble (CESM-DPLE), CESM Large Ensemble (CESM-LE) and the forced ocean–sea ice reconstruction.

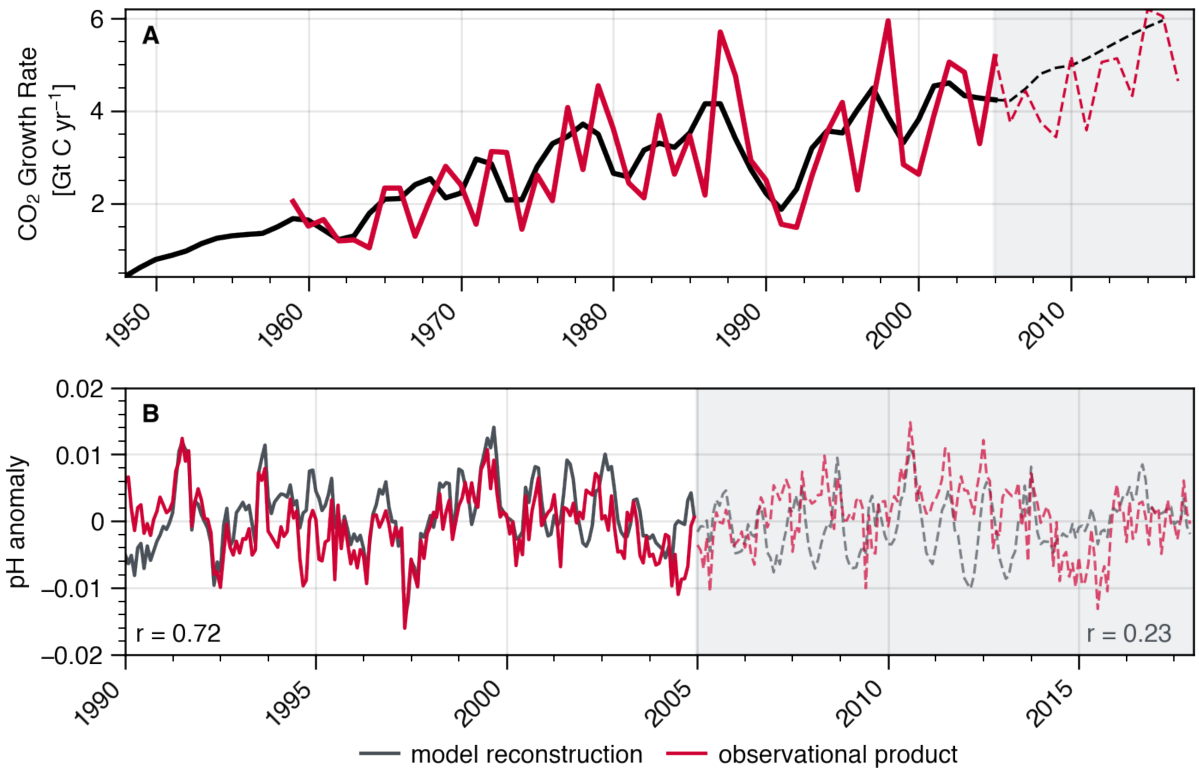

Supplementary Figure 1. *Growth rate of atmospheric CO_2 and surface pH anomalies.* (**A**) Global atmospheric CO_2 growth rate from the Global Carbon Project observations (red) and as used for external forcing in the reconstruction (black). (**B**) Surface pH anomalies (second-order polynomial fit and seasonal cycle removed) in the California Current System for the reconstruction (black) and observational product (red). The period following 2005 when the reconstruction switches to RCP8.5 forcing (*i.e.*, loses observed atmospheric variability in CO_2) is highlighted in gray. Correlation coefficients between the reconstruction and observational product are shown for the 1990-2005 period (bottom left) and 2006-2017 period (bottom right).

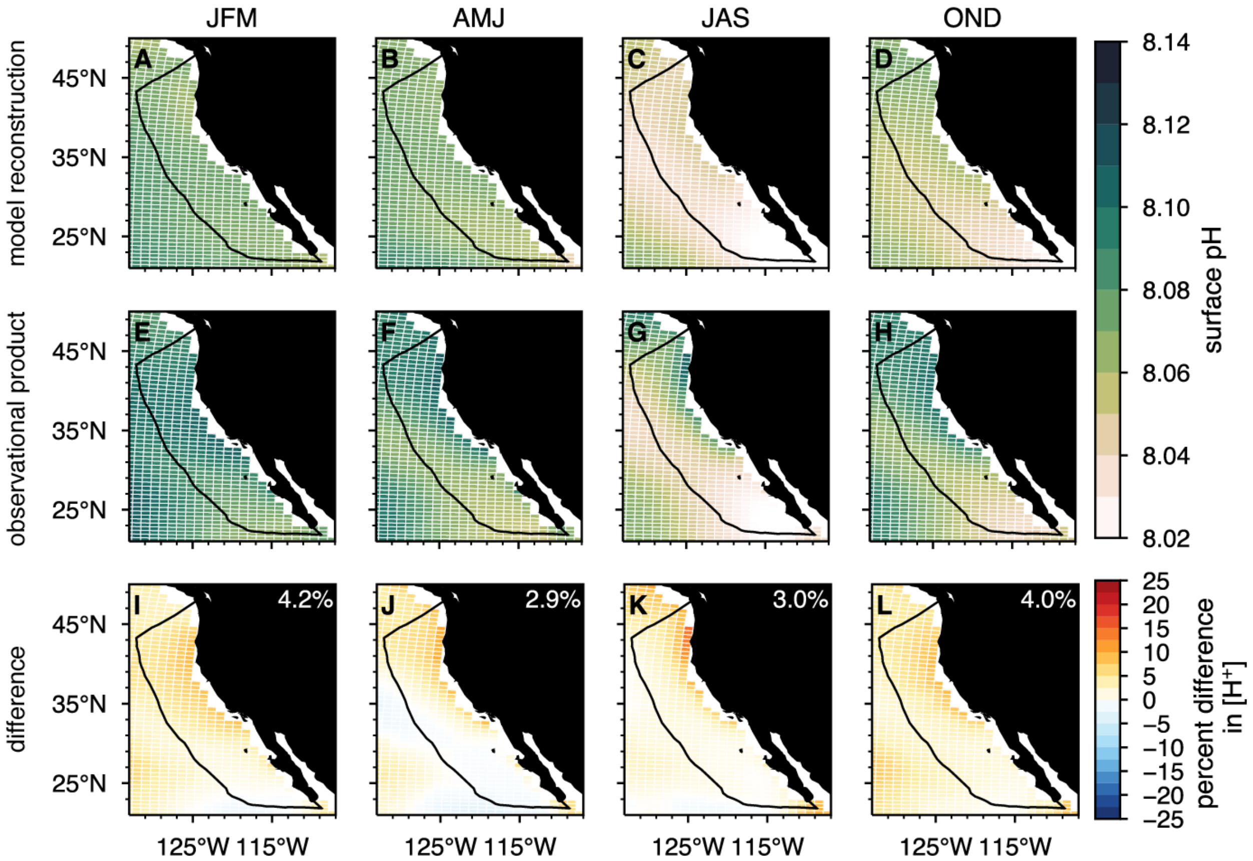

Supplementary Figure 2. *Spatial evaluation of surface pH in the model reconstruction*. (**A** to **D**) Seasonal climatology of surface pH for the reconstruction over 1990–2005. Here, the reconstruction is masked to match the missing coastal values in the observational product. (**E** to **H**) As in the previous row, but for the observational product. (**I** to **L**) Bias in the reconstruction as the percent difference in the surface hydrogen ion concentration (positive values indicate an acidic bias in the reconstruction). The percentage in the top right corner of (**I** to **L**) is the mean percent bias within the California Current Large Marine Ecosystem (black outline).

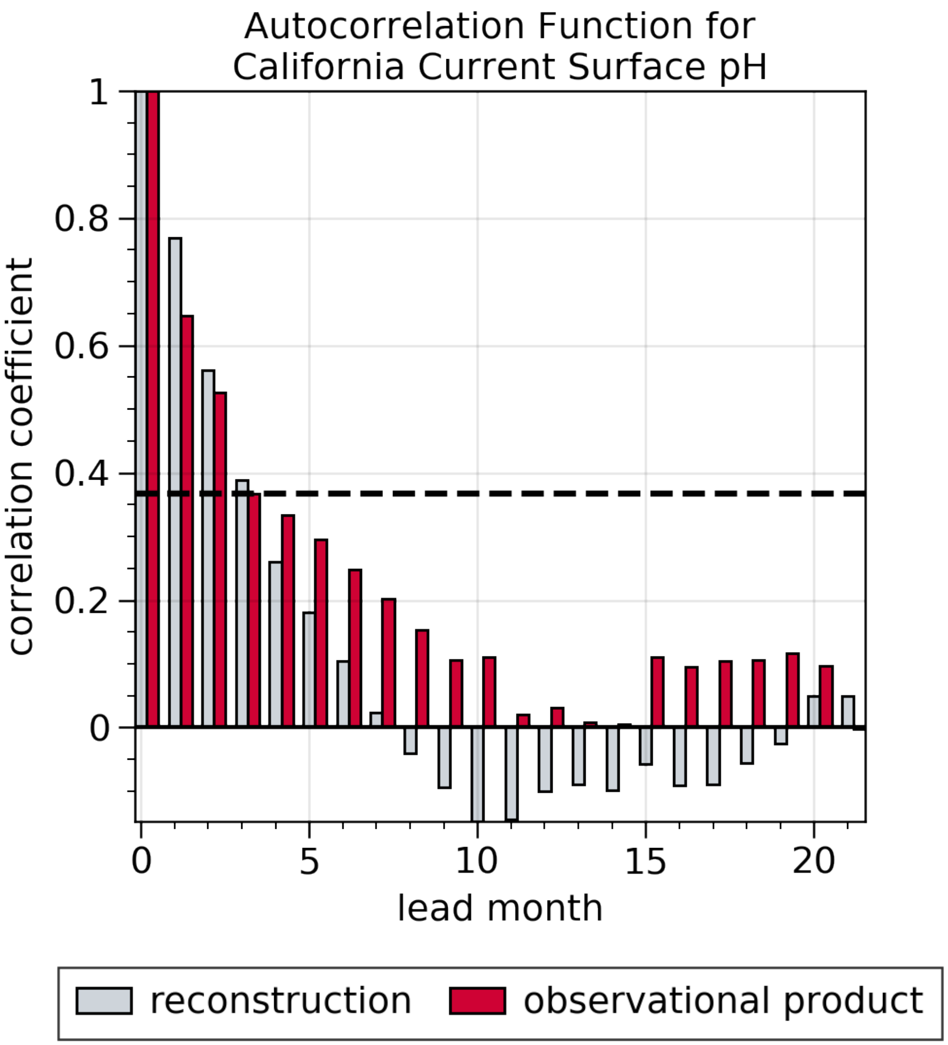

Supplementary Figure 3. *Autocorrelation of surface pH in the California Current.* Autocorrelation coefficients computed for monthly anomalies of surface pH in the California Current over 1990–2005, after removing a second-order polynomial fit and the monthly climatology. The black dashed line represents the e-folding level (1/e), which defines the boundary for the decorrelation time scale.

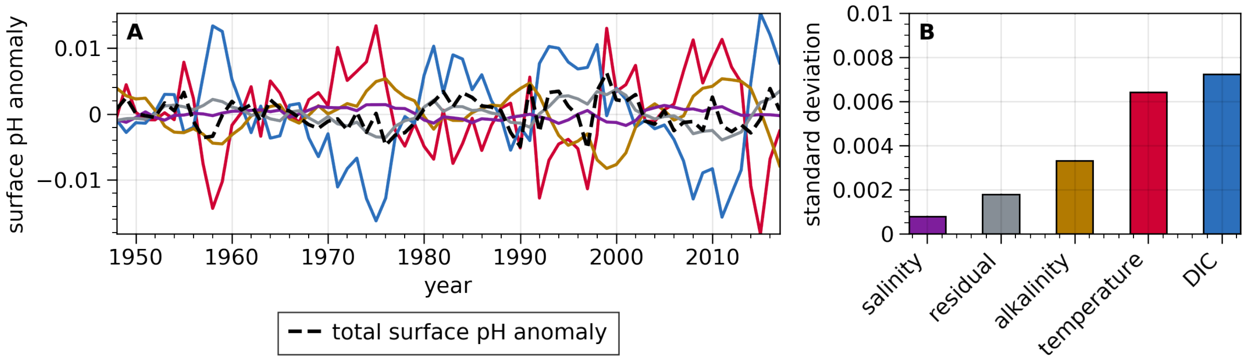

Supplementary Figure 4. *Drivers of surface pH variability in the California Current.* (**A**) Interannual variability in surface pH due to individual environmental variables (see Linaer Decomposition in Methods). Colors correspond to the color of each variable in (**B**). The dashed black line depicts the sum of all terms, or the total surface pH anomaly. (**B**) Magnitude of surface pH variability due to each environmental variable, quantified by the standard deviation from (**A**).

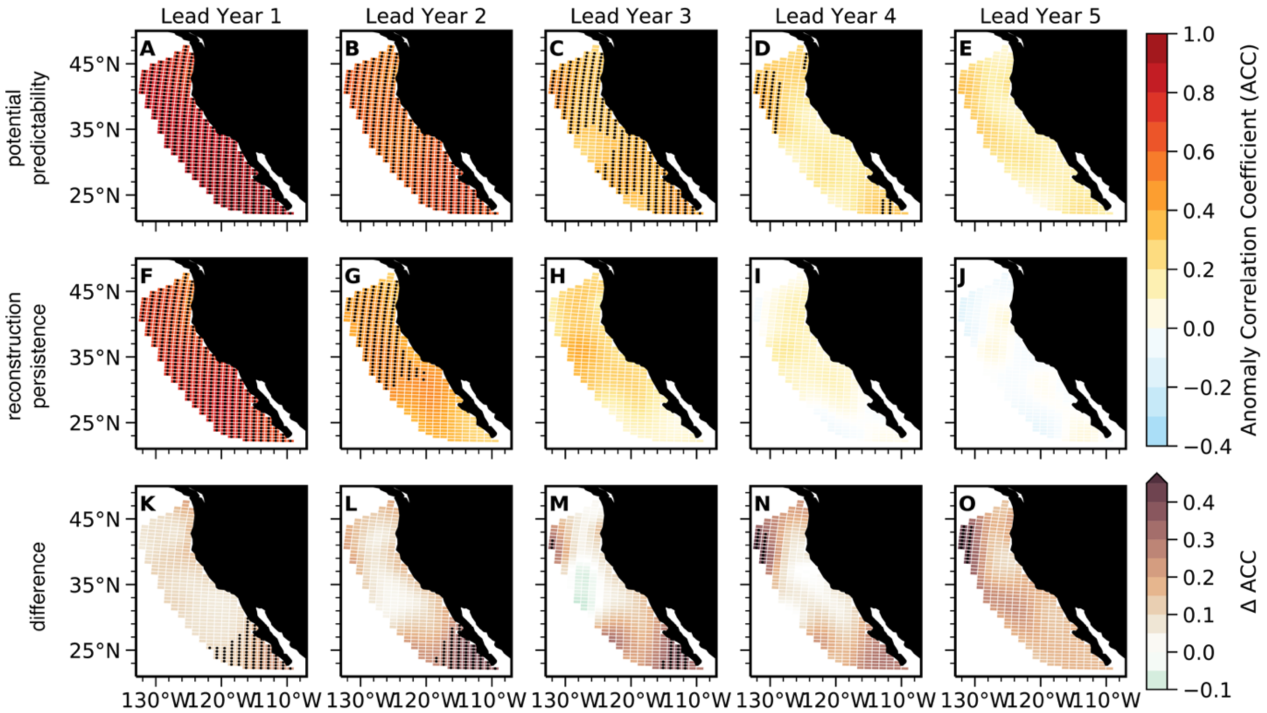

Supplementary Figure 5. *Potential predictability of dissolved inorganic carbon.* (**A** to **E**) Anomaly correlation coefficients (ACCs) for the Community Earth System Model Decadal Prediction Large Ensemble (CESM-DPLE) initialized forecasts of detrended annual surface salinity-normalized dissolved inorganic carbon (sDIC) anomalies for lead years one through five correlated with the model reconstruction. (**F** to **J**) Persistence forecast for the reconstruction for lead years one through five. Stippling in **A** to **J** denotes statistically significant correlations at the 95% level using a *t* test. An effective sample size is used in the *t* test to account for autocorrelation in the two time series being correlated[1]. (**K** to **O**) Difference between the CESM-DPLE forecast ACCs and persistence (ΔACCs). Stippling indicates that the initialized prediction is statistically significant over the persistence forecast at the 95% level using a *z* test. Only positive ACCs and ΔACCs are stippled.

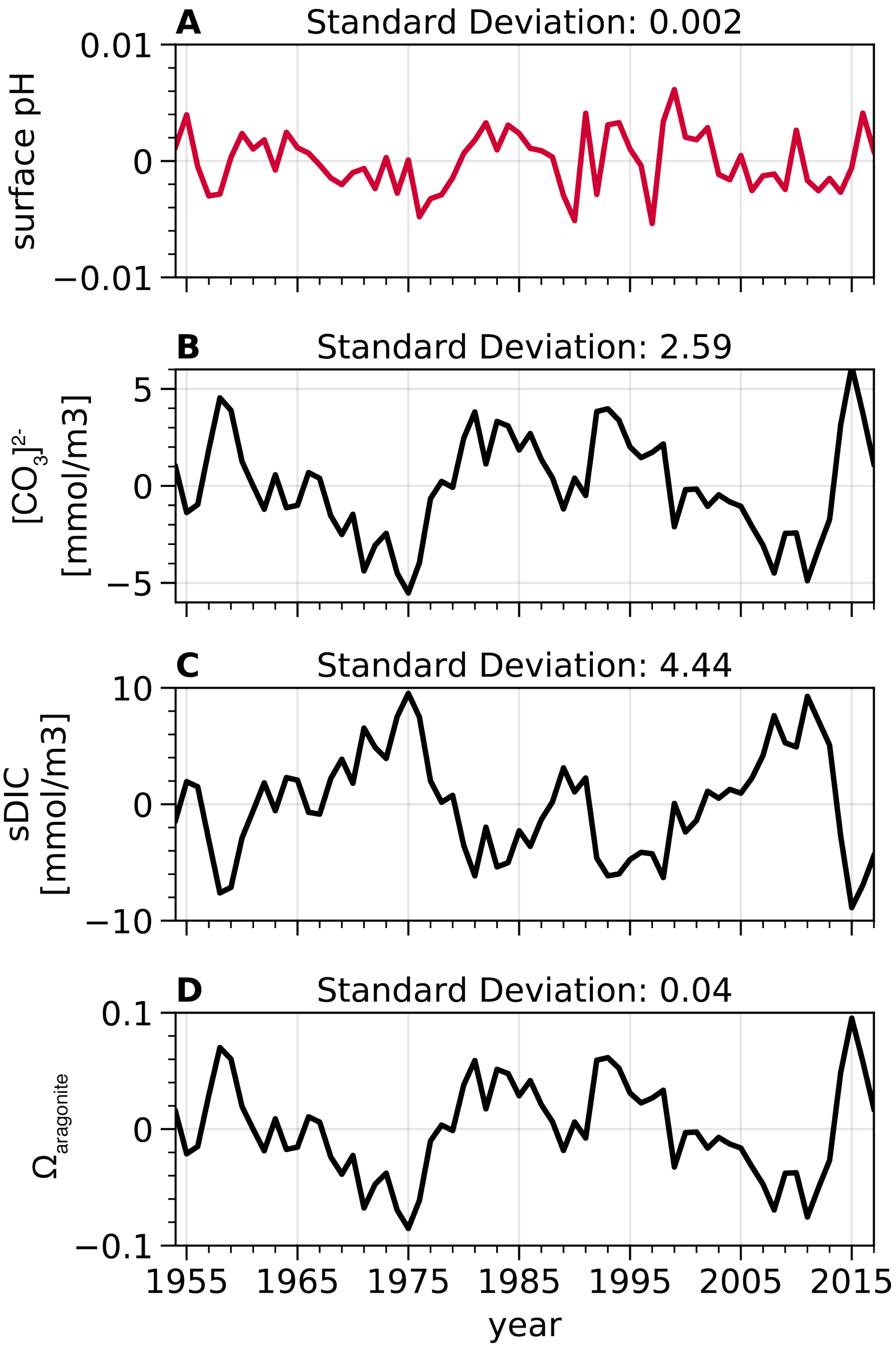

Supplementary Figure 6. *Variability of carbonate chemistry in the California Current.* (**A**) Area-weighted annual averages of surface pH in the model reconstruction in the California Current Large Marine Ecosystem after removing a second-order polynomial fit. (**B-D**) As in (**A**), but for the carbonate ion ($[CO_3]^{2-}$), salinity-normalized dissolved inorganic carbon (sDIC), and the aragonite saturation state ($\Omega_{aragonite}$), respectively.

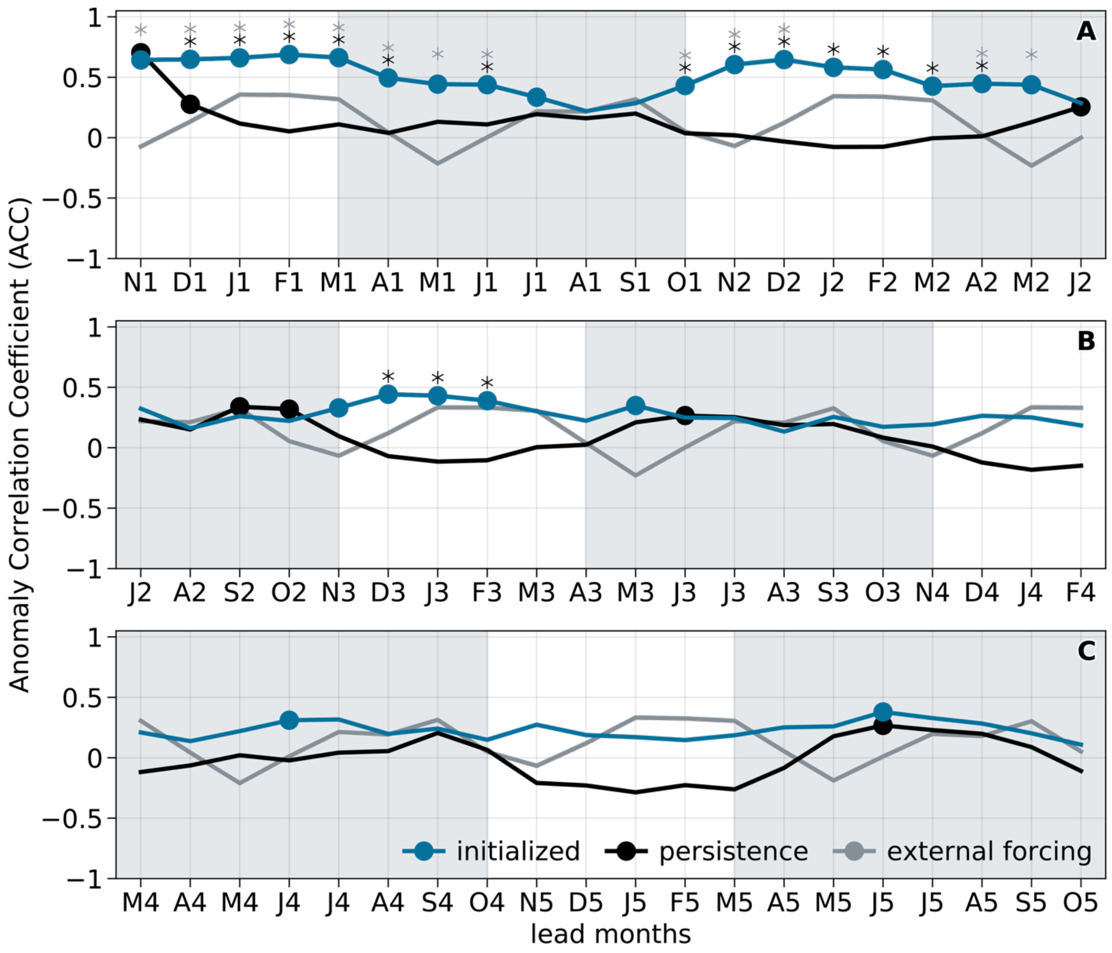

Supplementary Figure 7. *Domain-wide monthly predictability of surface pH anomalies.* Anomaly correlation coefficients (ACCs) for monthly surface pH predictions in the California Current Large Marine Ecosystem for the (blue) Community Earth System Model Decadal Prediction Large Ensemble (CESM-DPLE), (black) a persistence forecast from the reconstruction, and (grey) the uninitialized CESM Large Ensemble (CESM-LE). Filled circles denote statistically significant correlations at the 95% level using a *t* test. An effective sample size is used in the *t* test to account for autocorrelation in the two time series being correlated[1]. Black and gray asterisks indicate significant predictability over persistence and the uninitialized forecast at the 95% level using a *z* test, respectively. Grey-shaded regions indicate the approximate upwelling season. N1 indicates the November forecast for the monthly average of year one following initialization, for example.

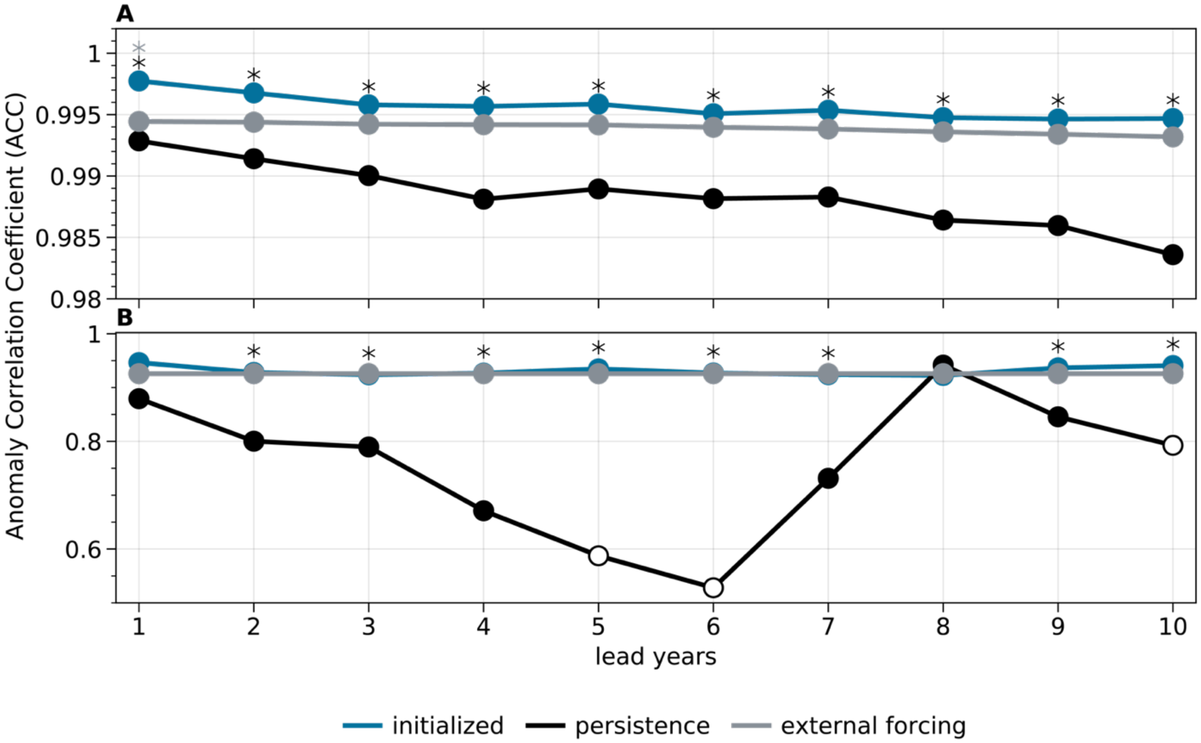

Supplementary Figure 8. *Domain-wide predictability of pH with acidification trend.* (**A**) Anomaly correlation coefficients (ACCs) for raw (no trend removed) surface pH forecasts relative to the model reconstruction over ten lead years for (blue) the Community Earth System Model Decadal Prediction Large Ensemble (CESM-DPLE), (black) a persistence forecast from the reconstruction, and (grey) the uninitialized CESM Large Ensemble (CESM-LE) ensemble mean. Filled circles denote statistically significant positive correlations at the 95% level using a *t* test. An effective sample size is used in the *t* test to account for autocorrelation in the two time series being correlated[1]. Black and gray asterisks indicate significant predictability over persistence and the uninitialized forecast at the 95% level using a *z* test, respectively. (**B**) As in (**A**), but for surface pH forecasts relative to the observational product.

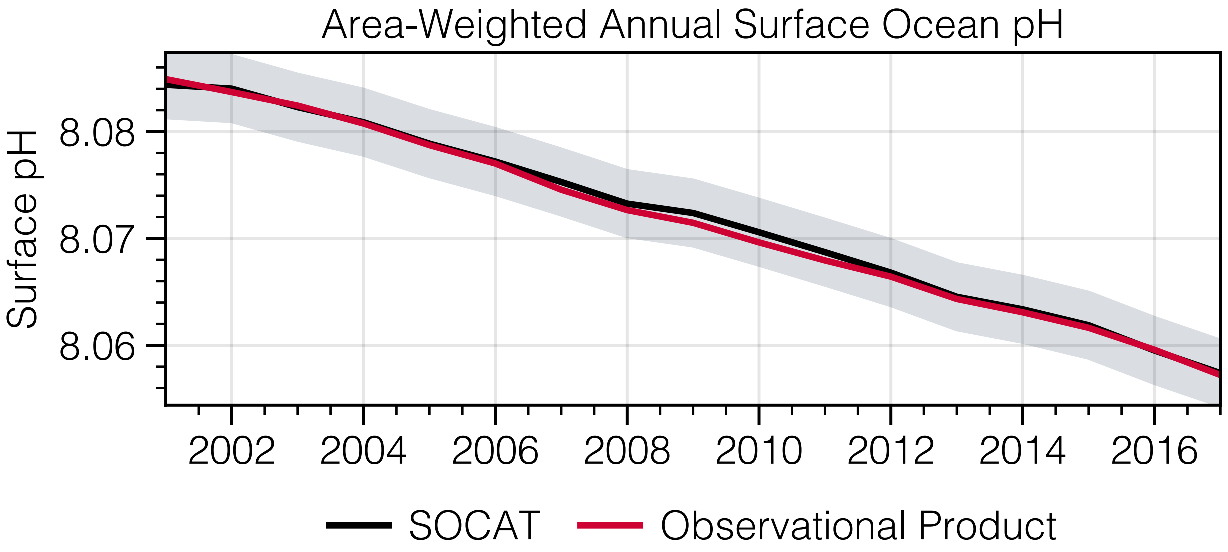

Supplementary Figure 9. *Validation of JMA observational product*. Global mean surface pH from the Surface Ocean CO_2 Atlas (SOCAT)-based product (black) and Japan Meteorological Agency (JMA) observational product (red) used in this study. Gray shading denotes uncertainty bounds provided with the SOCAT-based product.

Supplementary References

1. Yeager, S. G. *et al.* Predicting near-term changes in the Earth system: A large ensemble of initialized decadal prediction simulations using the Community Earth System Model. *Bull. Am. Meteorol. Soc.* **99**, 1867–1886 (2018).
2. Bretherton, C. S., Widmann, M., Dymnikov, V. P., Wallace, J. M. & Bladé, I. The effective number of spatial degrees of freedom of a time-varying field. *J. Clim.* **12**, 1990–2009 (1999).
3. Hurrell, J. W. *et al.* The Community Earth System Model: A framework for collaborative research. *Bull. Am. Meteorol. Soc.* **94**, 1339–1360 (2013).
4. Danabasoglu, G. *et al.* The CCSM4 oean component. *J. Clim.* **25**, 1361–1389 (2012).
5. Hunke, E. C., Lipscomb, W. H., Turner, A. K., Jeffery, N. & Elliott, S. CICE: the Los Alamos Sea Ice Model documentation and software user's manual vrsion 4.1 LA-CC-06-012. *T-3 Fluid Dyn. Group Los Alamos Natl. Lab.* **675**, (2010).
6. Lawrence, D. M. *et al.* Parameterization improvements and functional and structural advances in version 4 of the Community Land Model. *J. Adv. Model. Earth Syst.* **3**, (2011).
7. Moore, J. K., Lindsay, K., Doney, S. C., Long, M. C. & Misumi, K. Marine ecosystem dynamics and biogeochemical cycling in the Community Earth System Model [CESM1(BGC)]: Comparison of the 1990s with the 2090s under the RCP4.5 and RCP8.5 scenarios. *J. Clim.* **26**, 9291–9312 (2013).
